# Predicting diuretic efficiency in critically ill patients with acute decompensated heart failure: External validation of the BAN-ADHF score

**DOI:** 10.1016/j.ahjo.2026.100841

**Published:** 2026-07-24

**Authors:** Emmanuel Otabor, Justin Lam, Laith Alomari, Olayinka Afolabi-Brown, Kevin Bryan Lo

**Affiliations:** a Department of Medicine, Jefferson Einstein Philadelphia Hospital, Philadelphia, PA, USA; b Division Of Cardiovascular Disease, Jefferson Einstein Philadelphia Hospital, Philadelphia, PA, USA; c Division of Cardiovascular Medicine, Brigham and Women's Hospital, Harvard Medical School, Boston, MA, USA

**Keywords:** Acute heart failure, Diuretic resistance, Diuretic efficiency, Critical care, Intensive care, Risk prediction

## Abstract

In a critically ill cohort of 1505 patients with acute decompensated heart failure, the BAN-ADHF score was inversely associated with diuretic efficiency (Spearman ρ = −0.518) and discriminated the lowest-efficiency quintile (AUROC 0.780 at 24 h; 0.769 at 72 h), with efficiency falling more than four-fold across risk categories. This first external validation in an intensive-care population extends the evidence for BAN-ADHF to patients excluded from prior cohorts and supports prospective evaluation of score-guided decongestive strategies in critical illness.

Intravenous loop diuretics are central to decongestion in acute decompensated heart failure (ADHF), but inadequate response is common and associated with worse outcomes [1]. Segar et al. developed the eight-variable BAN-ADHF score to identify low diuretic efficiency, reporting an area under the receiver-operating-characteristic curve (AUROC) of 0.84 for the lowest efficiency quintile [2]. Prior validations were limited to trial or ward cohorts that excluded patients requiring intensive care, vasoactive support, or advanced circulatory therapies, leaving performance in critical illness uncertain [2], [3], [4].

We externally validated BAN-ADHF in the Medical Information Mart for Intensive Care IV database, version 3.1 [5]. Adults with ADHF in cardiac or medical intensive care units treated with intravenous loop diuretics were eligible. We excluded patients receiving chronic dialysis, those who died within 24 h, and those with incomplete score components (eFig. 1). Diuretic efficiency was urine output per milligram of intravenous furosemide equivalent over 24 and 72 h. Exploratory score categories were low (≤7), moderate (8–12), and high (≥13). We estimated 95% confidence intervals using 1000 bootstrap resamples. Baseline characteristics are shown in eTable 1.

The cohort included 1505 patients; 19.8% had cardiogenic shock, 38.7% required invasive mechanical ventilation, and 48.2% had chronic kidney disease. Among 1019 patients with calculable 24-hour efficiency, BAN-ADHF was inversely associated with efficiency (Spearman ρ = −0.518; 95% CI, −0.560 to −0.473) and identified patients in the lowest diuretic-efficiency quintile (AUROC, 0.780; 95% CI, 0.743–0.812). Median efficiency declined across low-, moderate-, and high-score categories from 47.4 to 29.0 to 11.3 mL/mg (*P* < 0.001; Fig. 1; eTable 2). Performance persisted at 72 h (ρ = −0.458; AUROC, 0.769; *n* = 781) and after excluding cardiogenic shock (AUROC, 0.773). Across heart-failure phenotypes, AUROCs ranged from 0.764 in reduced to 0.825 in preserved ejection fraction, although association strength varied across subgroups (eTable 3).

**Fig. 1:**
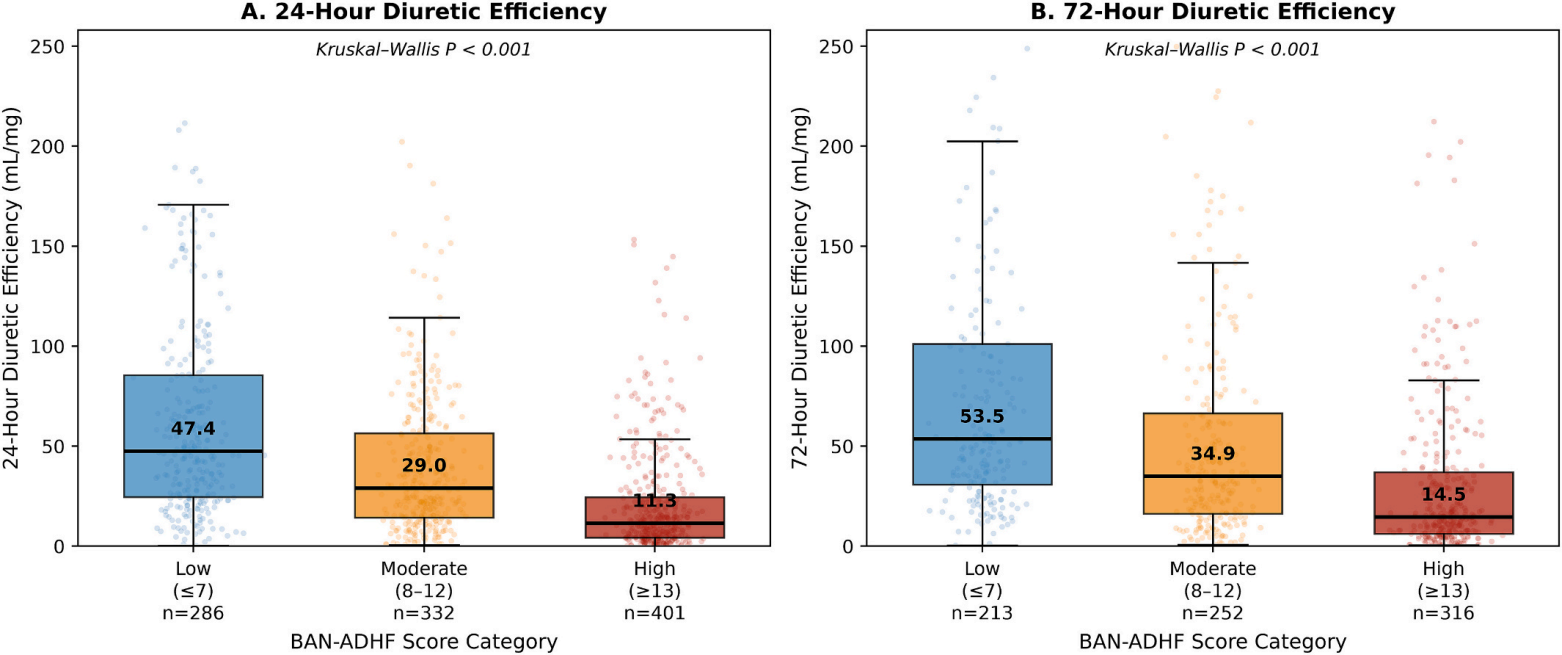
Diuretic efficiency by BAN-ADHF score category. Box plots of diuretic efficiency (mL urine output per mg intravenous furosemide equivalent) at (A) 24 h and (B) 72 h across low- (≤7), moderate- (8–12), and high- (≥13) score categories. Boxes denote the median (labeled) and interquartile range, whiskers the 1.5 × IQR range, and overlaid points individual patients; the number of patients per category is shown below each box. Median 24-hour efficiency fell 4.2-fold from the low- to the high-score category (Kruskal–Wallis *p* < 0.001). Score categories are exploratory (derived and evaluated in the same cohort). For visual clarity the y-axis is truncated at 250 mL/mg; 8 (24-hour) and 28 (72-hour) values above this range are not displayed but are included in all statistics.

At 72 h, the AUROC was 0.769, compared with 0.84 for the same lowest-quintile outcome in DOSE/ESCAPE [2] and 0.70 for a lowest-quartile outcome in CLOROTIC [3]. These findings extend prior ward-based validation, in which higher scores were associated with lower urine output despite greater diuretic doses [4], to a higher-acuity population. Applying these medians to 80 mg intravenous furosemide twice daily corresponds to approximately 7600 versus 1800 mL/day in low- versus high-score patients. This gradient may identify patients warranting closer monitoring and earlier reassessment of decongestive therapy.

Limitations include that MIMIC-IV contains data from a single center, potential selection bias from requiring an NT-proBNP measurement and complete score data, and a study period largely preceding widespread sodium-glucose cotransporter-2 inhibitor use. Because the exploratory categories were developed and evaluated in the same cohort, their gradient may be optimistic; the threshold-independent AUROC is more conservative. The retrospective design cannot establish whether score-guided treatment improves outcomes. The association weakened after excluding advanced chronic kidney disease (ρ = −0.323; eTable 2), possibly because renal function is an important determinant of both the score and diuretic response.

The BAN-ADHF score retained useful discrimination for diuretic efficiency in critically ill patients with ADHF. Prospective studies should determine whether score-guided monitoring or treatment improves decongestion in this population.

## CRediT authorship contribution statement

**Emmanuel Otabor:** Conceptualization, Formal analysis, Methodology, Writing – original draft, Writing – review & editing. **Justin Lam:** Writing – original draft. **Laith Alomari:** Formal analysis, Writing – original draft, Writing – review & editing. **Olayinka Afolabi-Brown:** Supervision, Writing – review & editing. **Kevin Bryan Lo:** Conceptualization, Methodology, Supervision, Writing – review & editing.

## Declaration of competing interest

The authors report no potential conflicts of interest. This research received no specific grant from funding agencies in the public, commercial, or not-for-profit sectors. MIMIC-IV is a publicly available, de-identified database; secondary analysis was exempt from institutional review board approval. Analytic code is available at https://github.com/nuel-otabormd/BAN-ADHF-Validation (archived at doi:https://doi.org/10.5281/zenodo.18124056).
