# Supplementary material for: Predicting diuretic efficiency in critically ill patients with acute decompensated heart failure: External validation of the BAN-ADHF score
Source: Am Heart J Plus. 2026 Jul 24;69:100841. doi: 10.1016/j.ahjo.2026.100841 (PMC13445343; doi:10.1016/j.ahjo.2026.100841)
Supplement: Supplementary file 1 [file mmc1.docx]

**Supplementary Material**

**Contents**

• eMethods. Cohort, diuretic-efficiency calculation, and risk-category derivation.

• eTable 1. Baseline characteristics by BAN-ADHF risk category.

• eTable 2. BAN-ADHF score performance for diuretic efficiency.

• eTable 3. Subgroup and heart-failure phenotype analyses.

• eFigure 1. Study cohort selection (CONSORT flow diagram).

• Data and code availability.

**eMethods**

**Data source and cohort**

We analyzed the Medical Information Mart for Intensive Care IV (MIMIC-IV), version 3.1, a publicly available, de-identified critical-care database. Adults (≥18 years) with a diagnosis of acute decompensated heart failure (ADHF) admitted to a cardiac or medical intensive care unit and treated with intravenous loop diuretics were eligible. We required an NT-proBNP measurement during hospitalization and an extractable medication-reconciliation section. Patients with end-stage renal disease receiving chronic dialysis, those dying within 24 hours of admission, and those with incomplete BAN-ADHF score components were excluded, yielding 1,505 patients; 1,019 had calculable 24-hour and 781 had calculable 72-hour diuretic efficiency. Data extraction was performed using Google BigQuery, and statistical analyses were conducted using Python 3.10.

**Diuretic efficiency**

Diuretic efficiency was defined as net urine output (mL) per milligram of intravenous furosemide-equivalent loop diuretic administered over the first 24 and 72 hours. Furosemide equivalents were calculated using established conversion ratios (1 mg oral bumetanide = 20 mg torsemide = 40 mg intravenous furosemide = 80 mg oral furosemide).

**Risk-category derivation**

The original BAN-ADHF derivation presented the score as a continuous variable (range 0 to 26) without categorical thresholds. To facilitate clinical application, we derived three risk categories (low, ≤7; moderate, 8 to 12; high, ≥13) using multiple statistical approaches applied to the 24-hour efficiency cohort (n=1,019). The high-risk threshold (≥13) showed strong convergence across methods, including the Youden index, a classification-tree split at 13.5, and the 67th percentile. It also matched all published frameworks (Alfonso, Pandey, and Mauch) and was adopted without modification. The low-risk threshold (≤7) was supported by Kruskal–Wallis optimization and the 25th percentile. Adjacent-score analysis showed a distinct efficiency drop between scores 7 and 8 (40.6 versus 33.2 mL/mg) but not between scores 6 and 7 (37.9 versus 40.6 mL/mg), indicating that score 7 behaves physiologically like low-risk scores. Classifying score 7 as low-risk reclassified 104 patients (6.9%) relative to the Alfonso framework and improved group separation (Kruskal–Wallis H, 229.8 versus 226.2). Because the categories were derived and evaluated within the same cohort, they should be regarded as exploratory. The threshold-independent area under the receiver-operating-characteristic curve (AUROC) provides the more conservative measure of performance.

**Statistical analysis**

Associations between the score and diuretic efficiency were assessed using Spearman (ρ) and Pearson (r) correlation coefficients. Discrimination was assessed using Harrell C-index for the continuous score and AUROC for classification of the lowest diuretic-efficiency quintile (Q20) and quartile (Q25). Efficiency across risk categories was compared using the Kruskal–Wallis test. All 95% confidence intervals were estimated from 1,000 bootstrap resamples.

**eTable 1. Baseline Characteristics by BAN-ADHF Risk Category**

| **Characteristic** | **Overall (n=1,505)** | **Low (n=446)** | **Moderate (n=480)** | **High (n=579)** |
| --- | --- | --- | --- | --- |
| Age, years | 70.0 ± 14.3 | 65.3 ± 15.5 | 72.3 ± 13.1 | 71.8 ± 13.3 |
| Male sex, n (%) | 791 (52.6) | 209 (46.9) | 243 (50.6) | 339 (58.5) |
| BAN-ADHF total score | 11.0 [7.0–15.0] | 5.0 [3.0–6.0] | 10.0 [9.0–11.0] | 16.0 [14.0–18.0] |
| Creatinine, mg/dL | 1.3 [1.0–1.9] | 0.9 [0.7–1.0] | 1.2 [1.0–1.5] | 2.0 [1.6–2.8] |
| BUN, mg/dL | 29 [20–48] | 17 [13–21] | 28 [22–37] | 50 [37–71] |
| NT-proBNP, pg/mL | 6,078 [2,141–14,788] | 2,078 [1,132–4,462] | 5,608 [2,458–11,348] | 14,107 [6,702–26,591] |
| Diastolic BP, mmHg | 68.2 ± 18.4 | 74.5 ± 18.2 | 67.2 ± 17.7 | 64.3 ± 17.8 |
| Home diuretic dose, mg/day † | 20 [0–80] | 0 [0–20] | 20 [0–40] | 80 [20–160] |
| Atrial fibrillation, n (%) | 897 (59.6) | 178 (39.9) | 294 (61.3) | 425 (73.4) |
| Hypertension, n (%) | 1,197 (79.5) | 272 (61.0) | 400 (83.3) | 525 (90.7) |
| Prior HF hospitalization, n (%) | 541 (35.9) | 66 (14.8) | 160 (33.3) | 315 (54.4) |
| LVEF, % ‡ | 40.1 ± 16.6 | 42.3 ± 17.1 | 41.2 ± 17.0 | 37.9 ± 15.7 |
| Diabetes mellitus, n (%) | 677 (45.0) | 154 (34.5) | 208 (43.3) | 315 (54.4) |
| Chronic kidney disease, n (%) | 725 (48.2) | 58 (13.0) | 202 (42.1) | 465 (80.3) |
| Cardiogenic shock, n (%) | 298 (19.8) | 49 (11.0) | 96 (20.0) | 153 (26.4) |
| Invasive mechanical ventilation, n (%) | 583 (38.7) | 181 (40.6) | 189 (39.4) | 213 (36.8) |

*Continuous variables are presented as mean ± SD or median [IQR]; categorical variables as n (%). †Oral furosemide equivalents. ‡LVEF was available in 934 patients (62.1%). Abbreviations: BP, blood pressure; BUN, blood urea nitrogen; HF, heart failure; IQR, interquartile range; LVEF, left ventricular ejection fraction; NT-proBNP, N-terminal pro-B-type natriuretic peptide.*

**eTable 2. BAN-ADHF Score Performance for Diuretic Efficiency**

| **Metric** | **Estimate (95% CI)** |
| --- | --- |
| 24-h efficiency, Spearman ρ | −0.518 (−0.560 to −0.473) |
| Continuous-score C-index | 0.677 (0.660–0.693) |
| AUROC, lowest 24-h quintile (≤7.5 mL/mg) | 0.780 (0.743–0.812) |
| AUROC, lowest 24-h quartile (≤9.9 mL/mg) | 0.772 (0.738–0.805) |
| 72-h efficiency, Spearman ρ | −0.458 (−0.516 to −0.402) |
| AUROC, lowest 72-h quintile (≤9.5 mL/mg) | 0.769 (0.730–0.805) |
| AUROC, lowest 72-h quartile (≤12.1 mL/mg) | 0.781 (0.748–0.815) |

*Median efficiency by score category is shown in the Figure. Category medians were 47.4, 29.0, and 11.3 mL/mg at 24 hours (Kruskal–Wallis H=229.8) and 53.5, 34.9, and 14.5 mL/mg at 72 hours (H=139.7); both p<0.001. Abbreviations: AUROC, area under the receiver operating characteristic curve; CI, confidence interval (1,000 bootstrap resamples). Sensitivity analyses: excluding cardiogenic shock, 24-hour AUROC was 0.773; excluding advanced chronic kidney disease (eGFR <30 mL/min/1.73 m²), ρ was −0.323 and AUROC 0.752, an expected attenuation given that creatinine is both a score component and an independent predictor of efficiency.*

**eTable 3. Subgroup and Heart-Failure Phenotype Analyses**

| **Subgroup** | **n** | **Spearman ρ** | **AUROC** | **p-interaction** |
| --- | --- | --- | --- | --- |
| Overall cohort | 1,019 | −0.518 | 0.780 | — |
| Age <65 years | — | −0.581 | — | 0.043 |
| Age ≥65 years | — | −0.482 | — | — |
| Diabetes | — | −0.600 | — | <0.001 |
| No diabetes | — | −0.432 | — | — |
| HFrEF (LVEF <40%) | 304 | −0.549 | 0.764 | — |
| HFmrEF (LVEF 40–49%) | 182 | −0.462 | 0.759 | — |
| HFpEF (LVEF ≥50%) | 162 | −0.542 | 0.825 | — |

*Consistency of BAN-ADHF score performance for 24-hour diuretic efficiency across pre-specified clinical subgroups and heart-failure phenotypes. Effect modification was tested using an interaction term; n and AUROC were not computed for the age and diabetes strata. Phenotype analyses were restricted to patients with a documented LVEF and calculable 24-hour efficiency. The em dash indicates not applicable or not reported. Abbreviations: AUROC, area under the receiver operating characteristic curve; HFmrEF, heart failure with mildly reduced ejection fraction; HFpEF, heart failure with preserved ejection fraction; HFrEF, heart failure with reduced ejection fraction; LVEF, left ventricular ejection fraction*

**eFigure 1. Study Cohort Selection**

*Flow diagram of study cohort selection from MIMIC-IV. Of 14,614 intensive-care admissions with an acute decompensated heart failure diagnosis, 1,505 met all inclusion criteria; 1,019 and 781 patients had calculable 24-hour and 72-hour diuretic efficiency, respectively. *Incomplete data denotes a missing BAN-ADHF score component, NT-proBNP measurement, or medication-reconciliation section. ADHF, acute decompensated heart failure; ICU, intensive care unit; IV, intravenous.*

**Data and Code Availability**

MIMIC-IV v3.1 is available to credentialed users via PhysioNet. Analytic code reproducing all results is available at https://github.com/nuel-otabormd/BAN-ADHF-Validation and archived at https://doi.org/10.5281/zenodo.18124056.
